# Decoding Fish Origins: How Metals and Metabolites Differentiate Wild, Cultured, and Escaped Specimens

**DOI:** 10.3390/metabo15070490

**Published:** 2025-07-21

**Authors:** Warda Badaoui, Kilian Toledo-Guedes, Juan Manuel Valero-Rodriguez, Adrian Villar-Montalt, Frutos C. Marhuenda-Egea

**Affiliations:** 1 Department of Biochemistry and Molecular Biology and Agricultural Chemistry and Edafology, University of Alicante, Carretera San Vicente del Raspeig s/n, 03690 Alicante, Spain; badaouiwarda34@gmail.com; 2 Department of Marine Sciences and Applied Biology, University of Alicante, Carretera San Vicente del Raspeig s/n, 03690 Alicante, Spain; ktoledo@ua.es (K.T.-G.); avmontalt@gmail.com (A.V.-M.); 3 Department of Biological Sciences, University of Bergen, P.O. Box 7803, 5020 Bergen, Norway; juanma.valero87@gmail.com

**Keywords:** fish traceability, aquaculture escapes, heavy metals, fatty acid profiling, NMR metabolomics, seafood authentication, environmental biomarkers, marine fish ecology

## Abstract

Background: Fish escape events from aquaculture facilities are increasing and pose significant ecological, economic, and traceability concerns. Accurate methods to differentiate between wild, cultured, and escaped fish are essential for fishery management and seafood authentication. Methods: This study analyzed muscle tissue from *Sparus aurata*, *Dicentrarchus labrax*, and *Argyrosomus regius* using a multiomics approach. Heavy metals were quantified by ICP-MS, fatty acid profiles were assessed via GC-MS, and metabolomic and lipidomic signatures were identified using 1H NMR spectroscopy. Multivariate statistical models (MDS and PLS-LDA) were applied to classify fish origins. Results: Wild seabream showed significantly higher levels of arsenic (9.5-fold), selenium (3.5-fold), and DHA and ARA fatty acids (3.2-fold), while cultured fish exhibited increased linoleic and linolenic acids (6.5-fold). TMAO concentrations were up to 5.3-fold higher in wild fish, serving as a robust metabolic biomarker. Escaped fish displayed intermediate biochemical profiles. Multivariate models achieved a 100% classification accuracy across species and analytical techniques. Conclusions: The integration of heavy metal analysis, fatty acid profiling, and NMR-based metabolomics enables the accurate differentiation of fish origin. While muscle tissue provides reliable biomarkers relevant to human exposure, future studies should explore additional tissues such as liver and gills to improve the resolution of traceability. These methods support seafood authentication, enhance aquaculture traceability, and aid in managing the ecological impacts of escape events.

## 1. Introduction

The Mediterranean Sea is characterized by a complex topography and dynamic meteorological patterns, making it especially vulnerable to climate-induced disturbances. Recent climatological studies report a growing prevalence of extreme warming events and sudden changes in water velocity, both of which are associated with global climate change and intensifying weather phenomena [1,2]. These shifts increase the risk of extreme marine events, such as explosive cyclones and structural damage to aquaculture systems, posing a threat to the sustainability of marine farming practices [3].

Fish account for approximately 17% of the global intake of animal protein and nearly 20% of daily protein consumption for over 3.2 billion people. Aquaculture now contributes nearly half of global seafood production and is continuing to expand faster than any other food sector. This rapid intensification has led to the widespread adoption of high-density farming systems, increasing vulnerability to disease outbreaks and structural failures [4,5].

Storm Gloria in 2020 caused widespread damage to aquaculture facilities along the Valencian and Murcian coasts, releasing thousands of cultured fish into the wild. Once escaped, these fish—reared under controlled conditions and fed formulated diets—exhibit physiological and behavioral differences from wild conspecifics [6]. Their presence in natural habitats may disrupt ecosystems through competition, disease transmission, and genetic introgression via interbreeding with native populations [5,7,8].

Fish escape incidents are generally categorized as routine (~5000 individuals/year), mass (up to 91× above normal), or catastrophic (up to 1800× above normal) events [9,10]. Escaped cultured fish can establish feral populations, disrupt native genetic pools, spread pathogens, and alter trophic dynamics. In the Mediterranean, such escapes have led to hybridization and resource competition with wild species, compromising ecosystem integrity. Commercial fisheries are also affected, as escaped fish mix with wild stocks, complicating assessments and lowering market value. While morphological traits—such as body roundness—may help to distinguish cultured fish, more robust biochemical tools are needed for accurate traceability [11,12].

Accurately identifying escaped fish once they enter natural ecosystems remains a key challenge for fishery management and seafood authentication. While morphological traits can be inconclusive, chemical markers such as heavy metals [13,14,15], fatty acids [6,16], and small-molecule metabolites [6,17,18] offer promising alternatives for origin tracing. Wild and cultured fish accumulate different biochemical signatures due to variations in diet, environment, and physiological adaptation.

In this context, we hypothesize that the integration of multiomics approaches—including metal analysis, fatty acid profiling, and 1H NMR-based metabolomics—can effectively differentiate wild, cultured, and escaped individuals among key Mediterranean species. The objectives of this study are (1) to quantify trace metals and assess their utility as origin markers; (2) to compare fatty acid profiles linked to feeding regimes; (3) to evaluate metabolomic and lipidomic signatures associated with environmental adaptation; and (4) to develop multivariate classification models to predict fish origin with a high accuracy.

This comprehensive approach will contribute to improved fishery management, aquaculture sustainability, and the prevention of seafood fraud.

## 2. Materials and Methods

### 2.1. Specimen Collection and Sample Preparation

Fish samples, including 30 gilthead seabream (*Sparus aurata*), 30 European seabass (*Dicentrarchus labrax*), and 20 meagre (*Argyrosomus regius*), were collected from fish markets, supermarkets, and local and wholesale markets in the Valencian Community and Murcia (Spain) between 2019 and 2022. Each specimen underwent an initial external examination to assess the presence of parasites, followed by biometric measurements and photographic documentation. Muscle tissue samples were subsequently extracted and stored at −20 °C until analysis. Based on their appearance, the presence of regenerated scales [9], and traceability references (‘commercial’ labelling), the fish were classified into the following origin groups: ‘Wild’, ‘Escaped’, and ‘Cultured’. Fish from the ‘Cultured’ group are easily identifiable, as they arrive at markets directly from marine aquaculture farms. Distinguishing between ‘Wild’ fish and ‘Escaped’ fish is much more complicated, as they arrive at fish markets after being caught by professional fishermen and, in principle, will all be wild. We now clarify that classification was based on geographic locations close to escape events and fish morphology typical of escaped individuals. The biometric data for the different fish species are shown in Table 1.

### 2.2. Heavy Metals Analysis by ICP-MS

Metal profiles were obtained from the digestion of muscle samples and ICP-MS analysis. These analyses were carried out at the Technical Research Services (SSTTI) of the University of Alicante. Fifteen trace elements selected on the basis of their frequency, bioaccumulation capacity, and potential detrimental effect on human health (Al, Cr, Mn, Fe, Co, Ni, Cu, Zn, As, Se, Mo, Cd, Tl, Pb, and Hg) were quantified. For chemical analysis, muscle samples were taken from the same individuals used in the previous biochemical analyses, i.e., 10 specimens per species (seabream, seabass, and meagre) and group of origin (wild, escaped, and cultured), except for wild seabass, for which sufficient specimens were unavailable during the escape event period.

The frozen samples were subjected to a digestion process in a solution of 4 mL of HNO_3_ and 0.5 mL of H_2_O_2_. The program consisted of different phases, reaching a final temperature of 240 °C over 45 min. Afterwards, the resulting sample was diluted to 15 mL with milli-Q water. Aliquots were taken and processed by standard ICP-MS analysis [19], extracting the concentrations of the different heavy metals listed previously.

### 2.3. Fatty Acid Analysis by GC-MS

The fatty acid profiles were constructed by extracting a battery of 35 of the most common fatty acids (Annex) by the direct extraction of fatty acid methyl esters (FAMEs) [20]. Analyses were conducted by the Technical Services of the Institute of Animal Science and Technology, Universitat Politècnica de València (UPV), which provided a quantitative description of the different fatty acids, as well as a chromatogram corresponding to each individual.

In the experimental process, muscle samples from the specimens used for metabolomic analysis were used. The experimental process consisted of direct FAME synthesis [20]. In detail, the frozen samples were divided into 0.5 g subsamples which were ground at room temperature between 10 and 15 s. The derivative was placed in Pyrex tubes with 1 mL of C13:0 standard (0.5 mg C13:0/mL MeOH); 0.7 mL of 10 N KOH in water; and 5.3 mL of MeOH. The tubes were incubated in a water bath at 55 °C for 1.5 h with 5 s of shaking every 20 min to dissolve and hydrolyze the sample. Subsequently, the samples were brought to room temperature by applying a cold bath, and 0.58 mL of 24 N H_2_SO_4_ in water was added. The tubes were mixed by inversion and incubated in water at 55 °C for an additional 1.5 h, shaking them gently for 5 s every 20 min. After a second cooling, 3 mL of hexane was added and homogenized in vortex for 5 min. Subsequently, the tubes were centrifuged for an additional 5 min. The hexane layers, containing the FAMEs, were transferred to gas chromatography vials and stored at −20 °C. Gas chromatographic (GC) analysis followed a standard protocol [20]. The GC-MS results are the concentrations of each fatty acid in mg per 100 g of sample.

### 2.4. 1H NMR Acquisition and Data Processing Parameters

A 500 µL sample was placed in a 5 mm NMR tube, and spectra were referenced to TSP at 0.00 ppm (polar samples) or chloroform at 7.26 ppm. All 1H NMR experiments were performed on a Bruker Avance 400 MHz (Bruker, Rheinstetten, Germany) equipped with a 5 mm HBB13C TBI probe with an actively shielded Z-gradient. The 1D solution-state 1H NMR experiments had a 2 s recycle delay, 32,768 time domain points, and a 2.556 s acquisition time. In total, 1024 scans were performed, and the experiment was conducted at 298 °K. Spectra were apodised through multiplication with an exponential decay, producing a 0.3 Hz line broadening in the transformed spectrum. The 1H NMR spectra were normalized and reduced to ASCII files using TopSpin (Bruker, Rheinstetten, Germany) and aligned using *icoshift* (version 1.0; available at https://ucphchemometrics.com/186-2/algorithms/ (accessed on 1 July 2025) [6]. The processing of 1H NMR spectra was performed in MATLAB (MathWorks, Natick, MA, USA). The region of water (4.60–4.95 ppm) and extreme high and low fields (<0.5 ppm and 10 ppm, respectively) were removed. Metabolites were identified in one-dimensional spectra using The Human Metabolome Database (HMDB, https://hmdb.ca/ (accessed on 1 July 2025)) and the literature cited in this study [6].

### 2.5. Statistical Analysis

Data from metal and fatty acids quantification were analyzed using Nonclassical Multidimensional Scaling (nMDS), specifically employing the Sammon mapping algorithm to visualize sample relationships based on pairwise similarity matrices. This method reduces dimensionality while preserving topological distances, emphasizing subtle group differences.

Metabolomic data (polar and apolar fractions) were organized into feature matrices and analyzed using Partial Least Squares–Linear Discriminant Analysis (PLS-LDA) in MATLAB (Math-Works, Natick, MA, USA) [21]. Pareto scaling was applied, and three components were used to build classification models. Model performance was evaluated through standard statistical parameters including R^2^X (explained variance in predictors), R^2^Y (explained variance in response), sensitivity, specificity, and area under the ROC curve (AUC).

## 3. Results

The present study evaluated fatty acid profiles and heavy metal concentrations as potential tracers for distinguishing escaped fish from wild and cultured counterparts. The findings revealed species-specific variations in metal accumulation and fatty acid composition, highlighting their potential use in traceability and food safety considerations.

**Figure 1:**
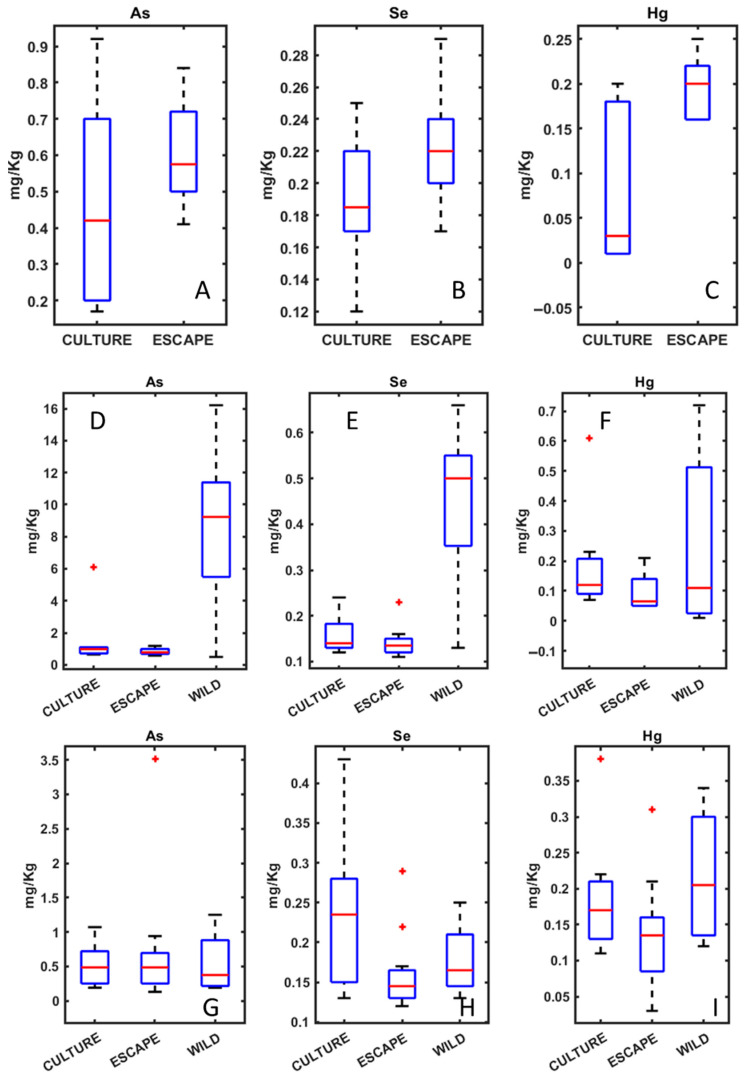
Boxplots of the concentrations of different metals (As, Se, and Hg) determined in meagre (**A**–**C**); in seabream (**D**–**F**); and in seabass (**G**–**I**).

The analysis of heavy metals in fish muscle tissue revealed significant differences between wild, escaped, and cultured fish (Figure 1). Using Inductively Coupled Plasma Mass Spectrometry (ICP-MS), we quantified the concentrations of 15 trace elements, including Al, Cr, Mn, Fe, Co, Ni, Cu, Zn, As, Se, Mo, Cd, Tl, Pb, and Hg. Among the species analyzed, only wild seabream exhibited higher levels of arsenic (As), selenium (Se), and mercury (Hg) compared to farm-raised fish.

**Figure 2:**
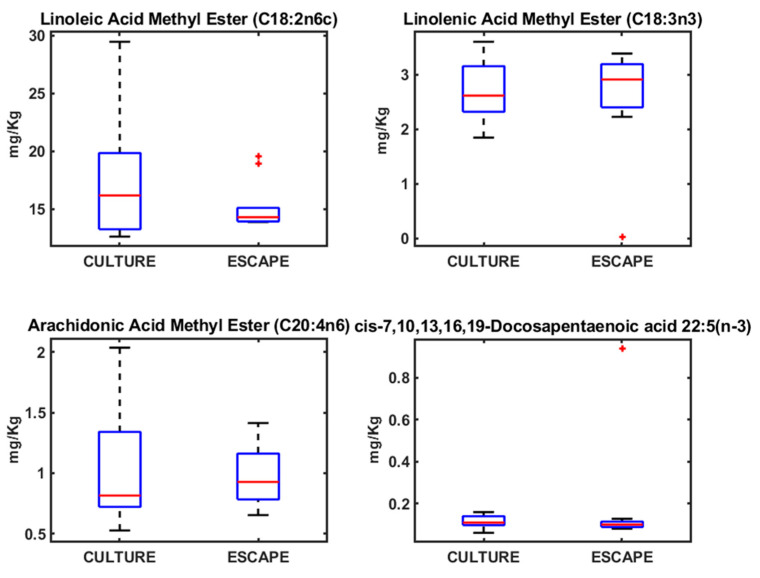
Boxplots of the concentrations of different AAGGs determined in meagre.

**Figure 3:**
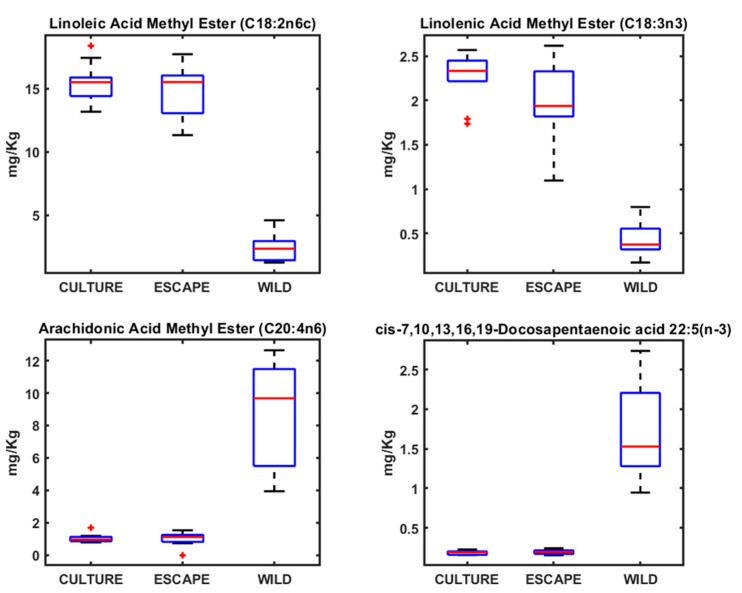
Boxplots of the concentrations of different AAGGs determined in seabream.

**Figure 4:**
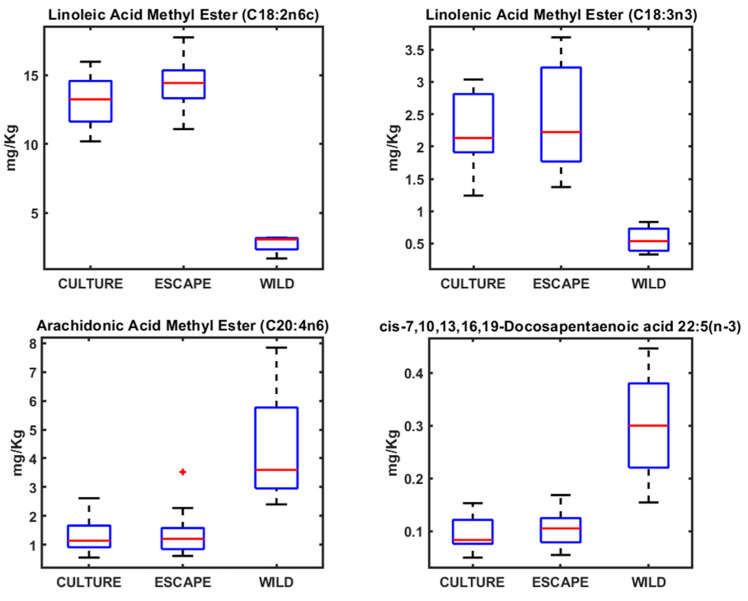
Boxplots of the concentrations of different AAGGs determined in seabass.

The fatty acid analysis yielded patterns consistent with the heavy metal findings, as both patterns respond to the feeding habits of each species and each situation (wild, cultured, and escaped) (Figure 2, Figure 3 and Figure 4). In the case of fatty acids, both seabream and seabass showed very similar patterns. With seabass and seabream, distinct lipid biomarkers enabled differentiation between wild, cultured, and escaped groups. Wild seabream and seabass displayed higher levels of arachidonic acid (C20:4n6) and docosapentaenoic acid (DHA; C22:5n-3) compared to cultured individuals (Figure 3 and Figure 4). These differences are linked to dietary variations, as wild seabream and seabass consume marine-derived lipids rich in long-chain polyunsaturated fatty acids (PUFAs), while cultured fish are fed diets containing vegetable oils [22,23].

Seabass, seabass, and meagre did not show significant differences in the fatty acid profiles between wild and cultured individuals, aligning with the findings from the heavy metal analysis. This lack of differentiation suggests that fatty acid composition alone may not serve as a reliable biomarker for traceability between species. However, in wild seabream and seabass, the normalization of fatty acid values improved classification, supporting the use of lipidomics as a tool for distinguishing fish origin (Figure 2, Figure 3 and Figure 4).

From a traceability perspective, seabream demonstrated the greatest potential for differentiation based on both heavy metal and fatty acid profiles (Figure 1 and Figure 3). The clear distinction between wild and cultured individuals highlights the feasibility of using these biomarkers in monitoring programs for escaped fish. With seabass, fatty acid patterns can also be used to identify the origin of the fish, as the differences between wild and cultured fish are clear, as is the case with seabream (Figure 3 and Figure 4). With meagre, there is no problem in identifying individuals caught by professional fishermen, as there is no wild meagre in this area of the Mediterranean. However, in areas where there is wild meagre, we will probably find a fatty acid pattern very similar to that found in wild seabream and seabass.

**Figure 5:**
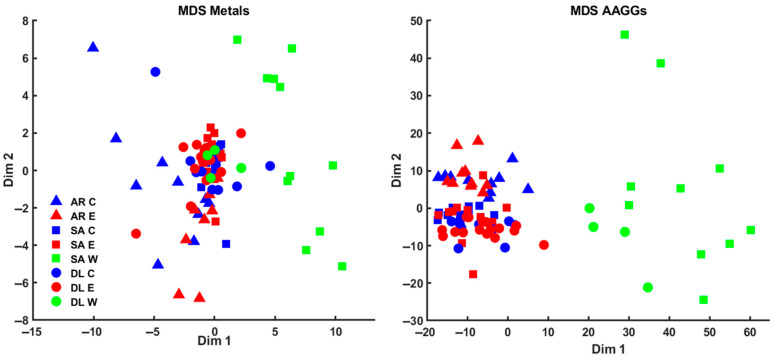
Multidimensional scaling approaches on the metals (stress of 0.1612) and the AAGGs (stress of 0.1226) obtained from the muscle of meagre, seabream, and seabass. The legend of the symbols is in the figure: AR (meagre), SA (seabream), DL (seabass), C (culture), E (escaped), and W (wild).

The data for the metals and AAGGS analyses were analyzed using the MDS method. Variations in the concentrations of metals and AAGGs in the different types of fish (cultured, escaped, and wild) can be visualized by Sammon mapping (Figure 5). Sammon mapping was used as this method diminishes the influence of large distances, which can completely dominate the map. The negative part of the x-axis in the Sammon mapping for metals was dominated by cultured and escaped fish samples, with wild fish samples dominating the positive part due to the higher concentrations of As, Se, and Hg in wild seabream (Figure 1). Seabream is a fish that consumes algae that accumulates these metals. The wild seabass samples, since they do not show differences in these metals, are placed next to the cultured and escaped fish (Figure 5). For Sammon’s map for AAGGs, we can see a similar distribution, but here, there are no wild seabass samples on the negative side, which are grouped with the wild seabream samples (Figure 5). The AAGG profiles are very similar in wild fish for both seabream and seabass (Figure 2, Figure 3 and Figure 4).

This study aimed to evaluate the utility of metabolomic and lipidomic profiling in distinguishing between cultured, escaped, and wild fish specimens, specifically focusing on seabass (*Dicentrarchus labrax*) and meagre (*Argyrosomus regius*). The metabolomic and lipidomic analyses revealed significant differences between groups, with particular emphasis on the roles of trimethylamine N-oxide (TMAO) and taurine as key biomarkers distinguishing wild fish from cultured and escaped fish [6,24,25,26,27].

**Figure 6:**
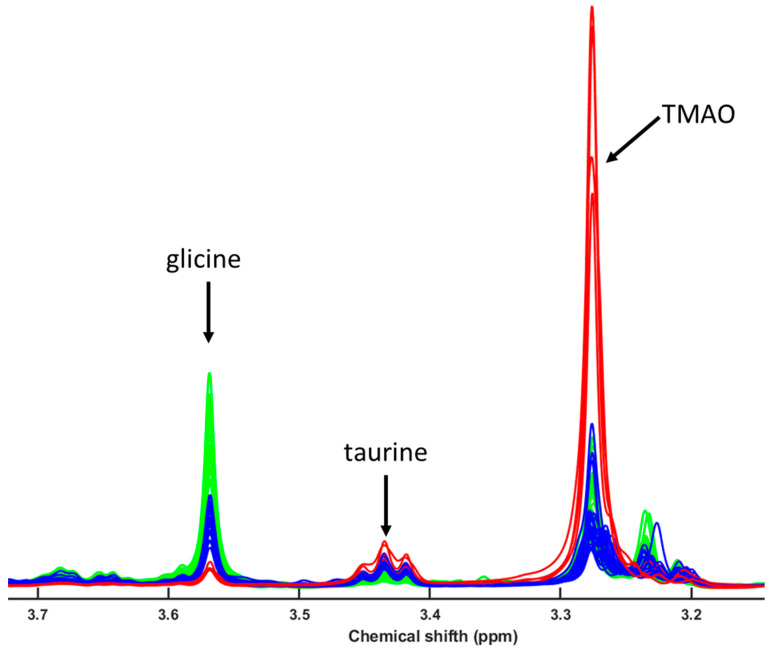
Magnified representation of the region of the 1D 1H-HRMAS NMR spectra of muscle tissue from wild seabass (red line) and cultured and escaped seabass (blue line), and cultured and escaped meagre (green line) with the signals of taurine (Tau) (S-CH_2_, 3.26), (TMAO (N-CH_3_, 3.28 ppm), taurine (Tau) (N-CH_2_, 3.42), and glicine (Gly) (CH, 3.56 ppm). The signals of TMAO and Gly are singlets, and the signals of Tau are triplets.

**Figure 7:**
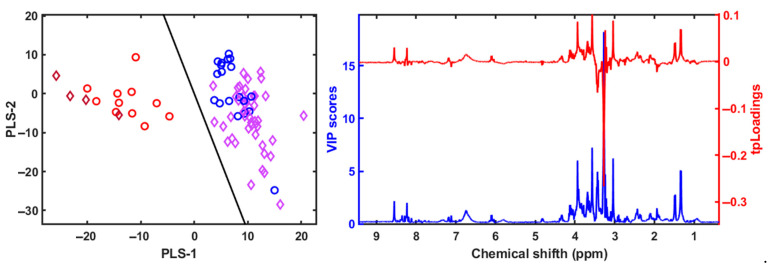
The PLS-LDA model score plots of 1H NMR spectra for the polar fraction of gilthead sea bream muscle samples (blue circles for escaped and cultured seabream, and red circles for wild seabream) was used for classification the spectra for the polar fraction for wild seabass (red diamond) and escaped and cultured seabass and meagre (purple diamond). The VIP scores and the pseudospectrum format PLS-LDA tpLoading was included [6]. The cumulative R2Y and R2X values for three variables were 0.88 and 0.50, respectively. The error was 0, the sensitivity was 1, the specificity was 1, and the AUC was 1.

The NMR-based metabolomic analysis of the polar fraction from seabass muscle revealed a clear separation between cultured and wild specimens, while escaped fish exhibited intermediate characteristics (Figure 6). The multivariate PLS-LDA analysis effectively grouped wild fish apart from the cultured and escaped seabream specimens [6]. We used the built model for seabream and tried to classify seabass and meagre specimens. We obtained an excellent classification of the samples (Figure 7), suggesting this as a good tool for traceability.

**Figure 8:**
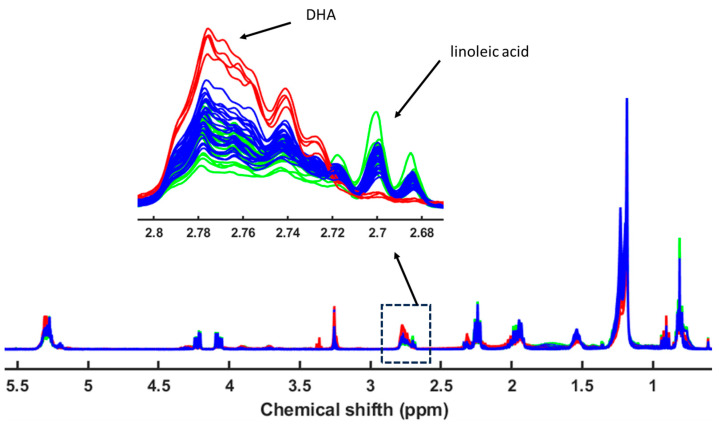
^1^H NMR spectra of the apolar fractions of wild seabass (red line), cultured and escaped seabass muscle samples (green line), and cultured and escaped meagre (blue line). The insert shows the enhanced linoleic acid region.

Lipid profiling using ^1^H NMR on the apolar fraction further confirmed dietary influences on metabolic composition. Cultured fish exhibited significantly higher levels of linoleic acid (C18:2n6) and α-linolenic acid (C18:3n3), characteristic of the vegetable oils used in aquafeeds. These fatty acids were absent in wild seabream and seabass, underscoring diet as a key determinant of lipid composition (Figure 8). Wild seabream and seabass showed higher concentrations of arachidonic acid (C20:4n6) and docosahexaenoic acid (DHA, C22:6n-3), derived from marine prey. These long-chain polyunsaturated fatty acids (LC-PUFAs) are essential for cell membrane function, neural development, and immune responses [28]. Their enrichment in wild seabream and seabass reflects their natural diet, rich in marine-derived lipids, in contrast to cultured fish, which receive diets supplemented with plant oils.

The distinct triplet signal at 2.79 ppm, corresponding to di-unsaturated fatty acids (DUFAs), was exclusively found in cultured and escaped fish (Figure 8). This signal was linked to linoleic acid, confirming its role as a dietary biomarker for aquaculture feeds. Adjusting the classification model to include this biomarker improved the differentiation of wild fish, validating its potential use in traceability applications [6].

**Figure 9:**
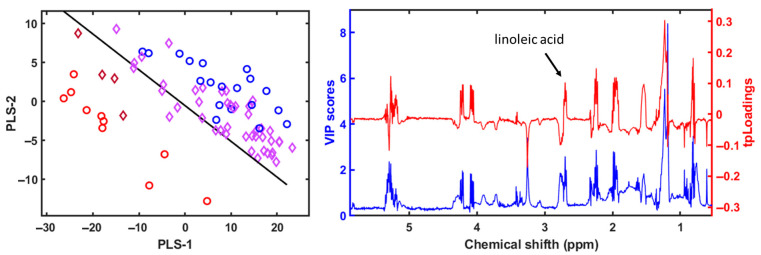
The PLS-LDA model score plots of 1H NMR spectra for the apolar fraction of gilthead sea bream muscle samples (blue circles for escaped and cultured seabream, and red circles for wild seabream) were used for classification of the spectra for the apolar fraction for wild seabass (red diamond) and escaped and cultured seabass and meagre (purple diamond). The VIP scores and the pseudospectrum format PLS-LDA tpLoading were included [6]. The cumulative R2Y and R2X values for three variables were 0.86 and 0.81, respectively. The error was 0, the sensitivity was 1, the specificity was 1, and the AUC was 1.

The PLS-LDA model created to classify the seabream specimens into cultured, escaped, and wild according to the 1H NMR spectra of the apolar metabolites [6] was used to classify the spectra obtained from the seabass and meagre samples (Figure 7). In this case, with the spectra obtained from the 1H NMR analysis of the apolar metabolites, we also obtained an excellent separation and classification of the seabass and meagre samples (Figure 9).

## 4. Discussion

### 4.1. Heavy Metals Analysis in Seabream, Seabass, and Meagre

The high concentration of As in seabream could be attributed to their natural diet, which includes benthic organisms and filter-feeding mollusks known for bioaccumulating arsenic. Notably, arsenic in wild seabream is primarily present in the form of arsenobetaine, which is considered non-toxic to humans [29]. The presence of arsenic in seafood has been widely studied due to its potential health risks, particularly when present in inorganic forms, which are highly toxic. However, marine organisms tend to accumulate organic arsenic forms, which have a lower toxicity. Despite this, continuous monitoring is required to assess the potential long-term effects of arsenic accumulation in marine food chains.

The metabolism of arsenic in aquatic organisms is a complex process influenced by environmental and biological factors. Arsenic exists in both inorganic (iAs) and organic (oAs) forms, with arsenobetaine (AsB) being the predominant non-toxic form in marine fish [29]. The biotransformation of arsenic involves redox reactions and methylation processes, which are mediated by microbial and enzymatic activity [30]. In aquatic environments, arsenic can induce oxidative stress by generating reactive oxygen species (ROS), which can damage cellular components such as lipids, proteins, and DNA [31]. Antioxidant enzymes, including superoxide dismutase (SOD) and glutathione peroxidase (GPx), play a crucial role in mitigating oxidative stress, highlighting the intricate balance between arsenic toxicity and cellular defense mechanisms [32]. Arsenic exposure triggers oxidative stress via reactive oxygen species (ROS). Superoxide dismutase (SOD) and glutathione peroxidase (GPx) form the first defense line by dismutating superoxide to hydrogen peroxide and reducing peroxides to water, respectively [33,34,35]. GPx, being selenium-dependent, is especially vital in wild fish with high Se and As levels; it prevents lipid peroxidation and cellular damage [15,30,32,36]. Recent findings highlight the multifaceted impact of arsenic and emerging contaminants on fish physiology and behavior. Experimental evidence shows that arsenic exposure increases aggression and bioaccumulation in fish, effects further exacerbated by the presence of aquatic algae. Concurrently, contaminants such as heavy metals and microplastics disrupt gut microbiota, leading to dysbiosis, oxidative stress, and compromised health [36,37].

Selenium is an essential micronutrient that plays a crucial role in antioxidant defense, immune function, and thyroid hormone metabolism [38]. Marine fish generally contain moderate to high selenium levels, which contribute to their nutritional value. Interestingly, selenium has been shown to counteract mercury toxicity by forming biologically inactive Se–Hg complexes, which reduce mercury’s bioavailability and toxicity [39]. The protective role of selenium is particularly relevant in wild seabream, where higher Se levels may mitigate the adverse effects of elevated mercury concentrations. However, excessive selenium intake can also be detrimental, leading to toxicity symptoms such as oxidative stress and metabolic disturbances. The balance between selenium and mercury is, therefore, an important factor in seafood safety and should be further investigated in future studies. Seleniumbinds strongly with mercury, forming insoluble Hg–Se complexes (e.g., Hg–Se–cysteine), thereby sequestering mercury and reducing its bioavailability to cellular proteins [34,40,41]. This conjugation also preserves selenium reserves for antioxidant selenoenzymes (like GPx), further mitigating Hg-driven oxidative damage.

Mercury levels were also elevated in wild seabream, likely due to bioaccumulation through the food chain, given that seabream is a carnivorous species. The trophic transfer of mercury from prey to predator results in higher concentrations in top consumers, which is a concern for both ecosystem health and human consumption. The observed differences in metal concentrations suggest that metal profiles can serve as biomarkers to differentiate wild seabream from cultured counterparts.

In contrast, seabass did not show significant differences in heavy metal content between wild and cultured groups. This similarity in metal accumulation suggests that cultured seabass diets may closely resemble those of wild seabass, leading to a homogenization of metal profiles. This observation limits the utility of heavy metals as a distinguishing factor for seabass traceability. Additionally, seabass are more pelagic feeders compared to seabream, potentially leading to a different bioaccumulation dynamic that reduces metal differentiation.

For meagre, which included only cultured and escaped groups, escaped specimens exhibited higher concentrations of As, Se, and Hg. These findings suggest that escaped fish consume wild prey, leading to a bioaccumulative effect similar to that observed in wild seabream. The absence of wild meagre in this region of the Mediterranean prevents direct comparisons, but the observed differences highlight the potential of heavy metal analysis in tracing fish escapes.

While this study focused exclusively on muscle tissue for heavy metal quantification, this choice was guided by its relevance to food safety and human exposure, as muscle is the primary edible portion of fish. Nevertheless, it is well established that other tissues—such as the liver, gills, and head—may serve as major sites of metal accumulation due to their roles in filtration, detoxification, and physiological regulation. For example, the liver typically exhibits higher concentrations of trace elements like cadmium and copper, reflecting its function in metal metabolism and storage. Future studies should consider a multi-organ approach to better understand the distribution of metals within fish and identify tissue-specific biomarkers of environmental exposure and origin. Such analyses would complement muscle-based assessments and could further enhance the resolution of traceability and ecotoxicological evaluations.

### 4.2. Fatty Acid Profiles in Seabream, Seabass, and Meagre

Fatty acids play fundamental roles in fish metabolism, influencing membrane fluidity, signaling pathways, and energy storage. DHA, for instance, is critical for neural development, vision, and immune function in fish. It is also a key component of phospholipids in cell membranes, enhancing membrane stability and function. Arachidonic acid (ARA) serves as a precursor for eicosanoids, which regulate inflammatory responses, immunity, and stress adaptation. Wild seabream and seabass’s elevated DHA and ARA levels reflect their natural diet, which provides a more balanced and diverse lipid profile compared to cultured fish [42,43,44].

Conversely, cultured seabream, seabass, and meagre exhibited elevated levels of linoleic acid (C18:2n6c) and linolenic acid (C18:3n3), consistent with the use of terrestrial plant-derived feed ingredients [42]. While these fatty acids can be elongated and desaturated to form long-chain PUFAs, their conversion efficiency in marine fish is relatively low. The higher prevalence of plant-derived fatty acids in cultured fish has implications for nutritional quality, as these lipids may alter the functional properties of cellular membranes and reduce the availability of essential fatty acids for physiological processes [45].

Linoleic acid (LA) and α-linolenic acid (ALA) are essential fatty acids of terrestrial plant origin, commonly found in aquaculture feeds. In contrast, long-chain polyunsaturated fatty acids (LC-PUFAs) such as docosahexaenoic acid (DHA) and arachidonic acid (ARA) are predominantly derived from marine sources and cannot be synthesized de novo by most fish species [46,47,48,49]. Consequently, the relative abundance of LA/ALA versus DHA/ARA in fish tissues serves as a reliable biomarker of dietary origin, distinguishing fish raised on plant-based feeds from those feeding on natural marine diets [24].

In wild fish, elevated levels of DHA and ARA are typically incorporated into membrane phospholipids, contributing to greater membrane fluidity and flexibility. These structural attributes enhance the lateral diffusion of membrane proteins such as G protein-coupled receptors (GPCRs), ion channels, and transporters, thereby promoting efficient cell signaling, nutrient uptake, and adaptive physiological responses [49,50]. Additionally, LC-PUFAs introduce kinks into phospholipid tails, reducing lipid packing density and increasing bilayer permeability, which facilitates thermal resilience and the dynamic remodeling of membranes under environmental stress [51,52].

In contrast, cultured fish tend to accumulate higher proportions of LA and ALA, which result in less fluid and more rigid membrane microdomains. This altered membrane composition may impair receptor mobility and diminish the efficiency of cellular signaling pathways involved in stress response, immunity, and growth regulation [53,54].

Moreover, LC-PUFAs play a critical role in modulating protein–lipid interactions within biological membranes. Their presence influences membrane thickness, protein docking sites, and the conformational dynamics of integral membrane proteins, affecting enzymatic activity and signal transduction processes [53,55,56]. Wild fish, with a higher DHA/ARA content, exhibit a more optimal integration of LC-PUFAs into membrane structures, supporting the proper function of membrane-associated proteins. In contrast, membranes of cultured fish may exhibit a reduced biochemical performance due to lower LC-PUFA incorporation.

Finally, while DHA and ARA in wild fish are primarily membrane-bound, in farmed fish, a substantial portion of lipids accumulates as neutral fat in cytoplasmic lipid droplets, particularly in muscle tissue. This reflects the high-lipid content of aquaculture diets and may further reduce the proportion of functional lipids incorporated into cellular membranes [24].

### 4.3. Metabolomic and Lipidomic Profiling Using NMR

A key finding was the significantly higher levels of TMAO in wild seabass compared to cultured and escaped specimens. TMAO plays a critical role in osmoregulation in marine fish, stabilizing proteins against denaturation and oxidative stress in varying salinity conditions [57,58]. Wild fish, exposed to fluctuating salinities, accumulate higher levels of TMAO, whereas cultured fish, kept under stable conditions, exhibit lower levels. High TMAO levels preserve protein folding and cellular integrity under pressure and salinity fluctuations, enhancing metabolic resilience—especially relevant in wild fish facing variable marine conditions [27,59]. Escaped fish had intermediate levels, suggesting partial metabolic adaptation but not full alignment with wild conditions. Studies indicate that TMAO enhances mitochondrial efficiency, contributing to an improved energy metabolism in wild fish [60]. Additionally, wild fish tend to have a more diverse gut microbiota, which enhances TMAO biosynthesis. In contrast, cultured fish, often exposed to antibiotic treatments, exhibit a reduced microbial diversity, limiting their ability to produce TMAO [57]. These findings are consistent with recent studies on gilthead seabream (Sparus aurata), which also reported significantly elevated TMAO in wild fish, indicating its role as a robust metabolic marker of environmental adaptation [27,60,61,62,63]. In aquaculture, lower TMAO levels might indicate reduced adaptation to natural environmental conditions and could be linked to inferior muscle quality. Furthermore, TMAO levels in wild fish have been correlated with increased muscle firmness, a key quality attribute for seafood consumers [61].

For meagre, a similar pattern emerged, with TMAO serving as the primary distinguishing metabolite. The intermediate TMAO levels in escaped meagre reinforce the idea that metabolic adaptation to the wild is an ongoing, time-dependent process [61].

As noted above, taurine also has higher levels in wild fish than in cultured and escaped fish from marine aquaculture stations (Figure 6 and Figure 7). This metabolite is very important in the process of osmoregulation, in membrane integrity, and in energy, amino acid, protein, and lipid metabolism, as well as in growth stimulation and antioxidative processes [59,64,65,66,67]. Deficiency in this nutrient causes serious physiological problems, which is why supplementation in aquaculture feed has been the subject of several studies, and elevated taurine in wild fish suggests improved osmoregulatory homeostasis compared to cultured fish. However, in our work, we still found significant differences in taurine levels between wild and farm-raised fish (Figure 7). Not only is the identification of taurine as a possible biomarker indicating the origin of the fish important, but further research is needed to investigate how low taurine levels affect the health of fish reared in marine aquaculture stations compared to wild fish [65,68].

Furthermore, cultured fish showed elevated levels of betaine, creatine, and glucose, reflecting metabolic adaptations associated with captivity. These metabolites, involved in energy storage and anaerobic metabolism, highlight the restricted mobility and high-energy diets typical of aquaculture environments [64,69,70]. The higher lactate levels in escaped fish suggest increased muscular activity, supporting the hypothesis of a metabolic shift following escape.

The fatty acid composition in fish muscle reflects not only dietary influences, but also physiological adaptations to energy metabolism, immune function, and stress resilience. DHA, for instance, is a crucial component of neuronal and retinal membranes, playing a fundamental role in cognitive function and vision in fish [71,72]. Meanwhile, arachidonic acid (ARA) acts as a precursor for eicosanoids, which modulate inflammatory responses and immune defense [48].

Fish raised in aquaculture systems often have lower DHA and ARA levels due to their plant-based diets, which lack these essential lipids. This may have implications for immune competence, as studies have shown that fish with higher dietary ARA intake exhibit enhanced stress tolerance and disease resistance [42].

## 5. Conclusions

This study proposes an integrated strategy combining heavy metal analysis, fatty acid profiling, and 1H NMR-based metabolomics to differentiate wild, cultured, and escaped fish in three Mediterranean species (*Sparus aurata*, *Dicentrarchus labrax*, and *Argyrisimus regius*) Heavy metals, particularly arsenic (As), selenium (Se), and mercury (Hg), were significantly elevated in wild and gilthead seabream, supporting their potential as traceability biomarkers. These differences are linked to natural feeding habits and environmental exposure. Fatty acid composition revealed species-specific patterns, with wild seabream and seabass showing higher levels of DHA and ARA, and cultured seabream, seabass, and meagre presenting elevated linoleic acid. These differences reflect dietary sources and feeding regimes. Metabolomic analysis, especially the quantification of TMAO, taurine, creatine, and betaine, provided robust discrimination between groups. TMAO emerged as a key marker of environmental adaptation and was consistently higher in wild fish. Escaped specimens displayed intermediate metabolic profiles, reflecting partial adaptation to natural conditions.

The classification models achieved a high accuracy, supporting their utility in seafood authentication. However, further validation is needed across broader geographic contexts and aquaculture systems. This study is restricted to three species and a specific Mediterranean region, which may limit its generalizability. Future directions include integrating compound-specific isotope analysis and microbiome profiling to enhance the resolution of traceability tools and support the real-time monitoring of aquaculture escape events.

A key limitation of this study is the exclusive analysis of muscle tissue for metal quantification. Although this approach is directly relevant to food safety and consumer exposure, it may not fully capture systemic patterns of metal accumulation. Tissues such as the liver, gills, and head are known to accumulate higher concentrations of certain metals due to their roles in detoxification, filtration, and metabolic regulation. Therefore, future research should adopt a multi-organ approach to enhance traceability resolution and identify tissue-specific biomarkers of environmental exposure and physiological adaptation.

## Figures and Tables

**Table 1:** Mean (± SD) biometric parameters of different fish species, including total weight, gutted weight, total length, and furcal length.

Species	Total Weight (g)	Gutted Weight (g)	Total Length (cm)	Furcal Length (cm)
Cultured Seabream	504.96 ± 113.17	461.61 ± 101.32	30.91 ± 3.81	26.14 ± 2.82
Escaped Seabream	606.39 ± 299.26	563.05 ± 273.57	30.90 ± 3.66	27.33 ± 3.76
Wild Seabream	347.69 ± 263.24	320.77 ± 250.79	28.26 ± 5.13	23.29 ± 4.70
Cultured Seabass	618.33 ± 212.59	563.66 ± 218.34	38.30 ± 4.40	33.22 ± 4.00
Escaped Seabass	869.64 ± 354.98	753.36 ± 320.69	41.81 ± 5.34	36.83 ± 4.63
Wild Seabass	597.08 ± 227.84	535.27 ± 211.42	37.38 ± 5.32	32.00 ± 4.27
Cultured Meagre	1274.93 ± 374.01	1198.78 ± 338.38	49.92 ± 4.47	45.34 ± 4.08
Escaped Meagre	1364.59 ± 389.41	1204.92 ± 528.79	52.58 ± 5.05	48.14 ± 4.85

## Data Availability

The data presented in this study are openly available in Marhuenda, Frutos (2024), “Decoding Fish Origen new”, Mendeley Data, V2, doi: 10.17632/6f2x25fyyz.1.
